# Temporal attending and prediction influence the perception of metrical rhythm: evidence from reaction times and ERPs

**DOI:** 10.3389/fpsyg.2015.01094

**Published:** 2015-07-29

**Authors:** Fleur L. Bouwer, Henkjan Honing

**Affiliations:** Amsterdam Brain and Cognition, Institute for Logic, Language and Computation, University of AmsterdamAmsterdam, Netherlands

**Keywords:** rhythm, meter, attention, prediction, MMN, P1, N1, music

## Abstract

The processing of rhythmic events in music is influenced by the induced metrical structure. Two mechanisms underlying this may be *temporal attending* and *temporal prediction*. Temporal fluctuations in attentional resources may influence the processing of rhythmic events by heightening sensitivity at metrically strong positions. Temporal predictions may attenuate responses to events that are highly expected within a metrical structure. In the current study we aimed to disentangle these two mechanisms by examining responses to unexpected sounds, using intensity increments and decrements as deviants. Temporal attending was hypothesized to lead to better detection of deviants in metrically strong (on the beat) than weak (offbeat) positions due to heightened sensitivity on the beat. Temporal prediction was hypothesized to lead to best detection of increments in offbeat positions and decrements on the beat, as they would be most unexpected in these positions. We used a speeded detection task to measure detectability of the deviants under attended conditions (Experiment 1). Under unattended conditions (Experiment 2), we used EEG to measure the mismatch negativity (MMN), an ERP component known to index the detectability of unexpected auditory events. Furthermore, we examined the amplitude of the auditory evoked P1 and N1 responses, which are known to be sensitive to both attention and prediction. We found better detection of small increments in offbeat positions than on the beat, consistent with the influence of temporal prediction (Experiment 1). In addition, we found faster detection of large increments on the beat as opposed to offbeat (Experiment 1), and larger amplitude P1 responses on the beat as compared to offbeat, both in support of temporal attending (Experiment 2). As such, we showed that both temporal attending and temporal prediction shape our processing of metrical rhythm.

## Introduction

In musical rhythm, we often perceive hierarchically organized regular salient moments in time, in the form of a metrical structure. The most salient level of a metrical structure is the beat or pulse. This is the regularity we usually tap and dance to. In addition, we can hear higher-level regularity, termed meter, in the form of alternating strong and weak beats. Metrical saliency often coincides with acoustic saliency in the form of an accent, but the relationship between the acoustic properties of music and the perceived metrical structure is not *per se* fixed (Large, 2008; Honing, 2013). When presented with an isochronous sequence of identical sounds, people perceive a pattern of alternating strong and weak tones, suggesting they induce a binary metrical structure from a rhythm that does not explicitly contain such a binary structure (Brochard et al., 2003; Abecasis et al., 2005; Potter et al., 2009). This phenomenon, known as subjective rhythmization or subjective accenting, is also a clear example of how a perceived metrical structure can influence the processing of rhythmic events. When listening to a rhythm with identical acoustic events, events in metrically strong positions (on the beat) can be perceived as louder than events in weaker positions (offbeat), even though all events are acoustically identical (Repp, 2010). In addition, a perceived metrical structure causes sound events to be more expected at metrically strong positions than at metrically weak positions (Ladinig et al., 2009). Two possible mechanisms underlying the influence of a perceived metrical structure on the processing of rhythmic events are temporal attending and temporal prediction.

The first mechanism, *temporal attending*^1^, is described by the Dynamic Attending Theory (DAT), a prominent theory of the perception of metrical structure. According to DAT, the perception of metrical structure is the result of regular dynamic fluctuations in attentional resources, peaking at metrically strong positions (Large and Jones, 1999; Jones, 2010). Entrainment of neural oscillations to regular rhythmic events has been suggested to underlie these fluctuations in attentional resources (Large, 2008). The availability of more resources at metrically strong positions is thought to cause a general heightened sensitivity for events at those positions. This heightened sensitivity on the beat is supported by studies looking at processing of temporal deviations (Large and Jones, 1999), pitch (Jones et al., 2002), and speech sounds (Quené and Port, 2005). In addition, electrophysiological studies using oddball paradigms have shown larger event-related potentials (ERPs) to unexpected silences or intensity decrements in metrically strong positions than in metrically weak positions (Potter et al., 2009; Bouwer et al., 2014).

Temporal fluctuations in attentional resources can also explain the occurrence of subjective accents in metrically strong positions. Attention has been proposed to enhance early sensory responses to sound (Lange, 2013). Electrophysiological studies show that auditory evoked potentials (AEPs) are enhanced for events in metrically strong positions as compared to for events in metrically weaker positions (Abecasis et al., 2009; Iversen et al., 2009; Schaefer et al., 2010; Tierney and Kraus, 2013). This is in line with more attentional resources being available in metrically strong positions than in metrically weak positions due to temporal attending and may cause events in metrically strong positions to be perceived as subjectively accented.

Recently, Vuust and Witek (2014) have proposed an alternative view on the perception of metrical rhythm, which emphasizes the importance of *temporal prediction*. They suggest that the perception of metrical rhythm can be explained within the framework of predictive coding (Clark, 2013). A metrical structure provides predictions about upcoming events and the degree to which these predictions are met provides a prediction error, which is used to update the perceived metrical structure. Like in DAT, within the framework of predictive coding, the perception of metrical rhythm is thought to be an interplay of top-down, endogenously driven, and bottom-up, exogenously driven processes (Vuust and Witek, 2014). However, the nature of the top-down, endogenous process differs between these two theories, with predictive coding stressing temporal prediction instead of temporal attending, which leads to different hypotheses about the influence of the metrical structure on the processing of rhythmic events (see Table 1).

**Table 1:** **Hypothesized effects of temporal attending and prediction on the detection of intensity increments and decrements in different metrical positions**.

**Mechanism**	**Hypothesis**	**Predicted experimental effect**			
		**Increments**		**Decrements**	
		**On the beat**	**Offbeat**	**On the beat**	**Offbeat**
Temporal attending	Heightened sensitivity on the beat	+	−	+	−
Temporal prediction	Better processing of events with large prediction error	−	+	+	−

“*+” indicates relatively improved detection; “−” indicates relatively impoverished detection*.

First, DAT predicts better detection of unexpected events in metrically strong than weak positions, due to heightened sensitivity at metrically strong positions. However, loud sounds are more expected in metrically strong positions than in metrically weak positions. As such, the prediction error for an unexpected intensity increment in a metrically weak position is likely bigger than for an unexpected intensity increment in a metrically strong position, which predicts better detection of the former than the latter. Thus, while temporal attending would lead to enhanced processing of any event in a metrically strong position, temporal prediction would lead to enhanced processing of metrically unpredicted events (cf. Clark, 2013). Indeed, several studies have found better detection of unexpected intensity increments in metrically weak than strong positions (Abecasis et al., 2009; Geiser et al., 2010), in line with temporal prediction but not temporal attending affecting the processing of rhythmic events.

Second, while attention is thought to enhance early responses to auditory events, prediction is thought to attenuate those responses (Schafer et al., 1981; Lange, 2013). In a study comparing the responses to regular and irregular sound sequences, Schwartze et al. (2013) found attenuation of the auditory P1 response to acoustic events in the regular sequences. Similarly, Sanabria and Correa (2013) showed that the auditory N1 response was attenuated for events presented after a predictable time-interval, but not for events presented after an unpredictable time-interval. These studies show that temporal predictability attenuates the response to acoustic events. As such, while temporal attending as proposed in DAT would lead to enhancement of responses to events at metrically strong positions, temporal prediction would lead to attenuation of these responses, as events in metrically strong positions are highly expected (Ladinig et al., 2009).

**Figure 1:**
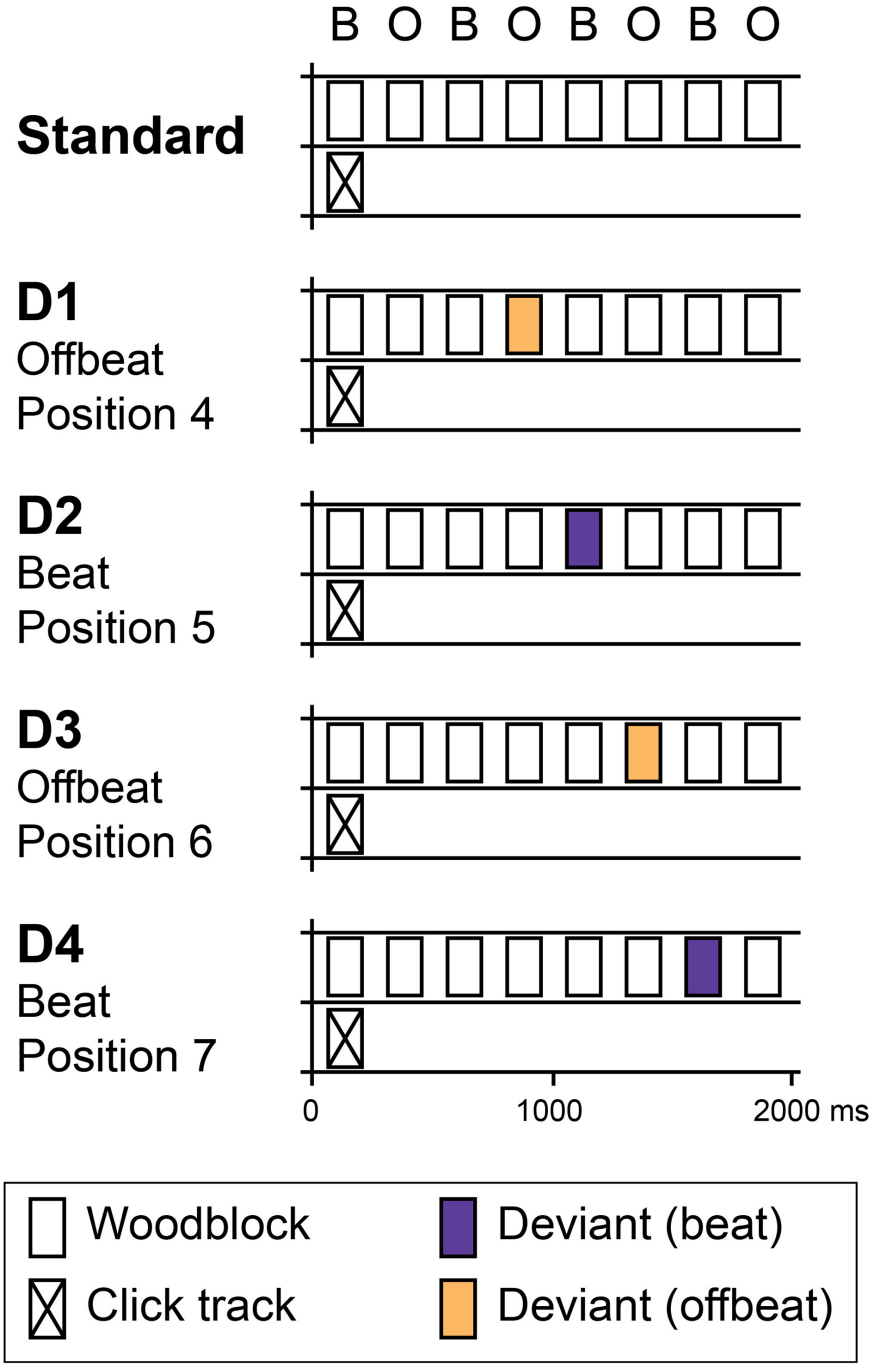
**Schematic overview of standard and deviant patterns**. Standards consisted of eight identical woodblock sounds with an inter-onset interval of 250 ms in which subjects were expected to perceive a binary pattern of alternating beats (B) and offbeats (O). Patterns were presented in a continuous stream. In position 1, a click track sound was superposed on the pattern to ensure phase alignment within the stream of rhythms. Four deviant patterns were used (D1–D4). In two patterns, deviants were introduced in offbeat positions (D1 and D3, positions 4 and 6 respectively). In two patterns, deviants were on the beat (D2 and D4, positions 5 and 7). At each position (D1–D4), two types of deviants were used: intensity increments and intensity decrements. In Experiment 1, deviants of three different magnitudes were used: 4, 6, and 9 dB. In Experiment 2, only 9 dB deviants were used.

In the current study, we aimed to examine the influence of temporal attending and temporal prediction to the processing of a metrical rhythm. To be able to disentangle the contributions of temporal attending and temporal prediction, we used an auditory oddball paradigm in which we introduced infrequent unexpected events in the form of both intensity increments and decrements at different metrical positions in an isochronous rhythm (see Figure 1). We expected the rhythm to induce a binary metrical structure, with odd positions being metrically strong (on the beat) and even positions metrically weak (offbeat, see Potter et al., 2009). To ensure that people heard the alternating strong and weak tones with the same phase, a click track sound was superposed on the isochronous rhythm every eight tones.

In Experiment 1, we used a speeded detection task in which participants were required to respond to the deviants. As described above, temporal attending is hypothesized to lead to better detection of deviants in metrically strong than weak positions. Temporal prediction is hypothesized to lead to better detection of increments in metrically weak than strong positions and better detection of decrements in metrically strong than weak positions, consistent with larger prediction errors for increments in weak positions and for decrements in strong positions. As such, while temporal attending and temporal prediction are hypothesized to have the same effect on the detection of decrements, the detection of increments differentiates between the presence of these two mechanisms (Table 1). It must be noted that temporal attending and temporal prediction may not be independent. Attending in time may lead to strong predictions about the occurrence of an event (Lange, 2013). If both temporal attending and prediction are present, their effects on the detection of increments may cancel each other out. The concurrent presence of both mechanisms would thus lead to large effects of metrical position on the detection of decrements and null or small effects of metrical position of the detection of increments.

In Experiment 2, we examined, using EEG, whether the influence of metrical structure on the processing of rhythmic events persisted with lower general levels of attentional resources directed at the rhythm. Previously, using ecologically valid stimuli in which acoustic saliency and metrical saliency always coincided, we did find differences in processing of unexpected events in metrically strong and weak positions, even when attention was directed elsewhere, showing that the induction of a metrical structure from exogenous cues is possible with lower levels of attentional resources (Bouwer et al., 2014). Contrary to this, Chapin et al. (2010) found that when listening to highly syncopated rhythms, attention was required to recruit the basal ganglia, which has been associated with the perception of metrical structure (Grahn and Brett, 2007; Grahn, 2009). It is unclear however, whether the lack of basal ganglia activity found by Chapin et al. (2010) when people were not attending to the rhythms was due to the highly syncopated nature of the rhythms or to the lack of acoustic salient accents indicating the metrical structure. We have suggested that the induction of a metrical structure from rhythms without clear acoustic accents may be possible with lower levels of attentional resources, as long as the metrical structure is sufficiently simple (Bouwer et al., 2014). In Experiment 2, we tested this hypothesis by examining the contributions of temporal attending and temporal prediction to the processing of metrical rhythm while attention was directed away from the rhythm.

Whereas in Experiment 1, reaction times and detection rates provided a direct measure of the detectability of the deviant events, in Experiment 2 we used the mismatch negativity (MMN) as an index of deviant detection. MMN is an ERP component that has been shown to occur without attention directed to a sound (Näätänen et al., 2007) and is affected by our predictions in the auditory modality (Winkler, 2007). As such, it is a very useful instrument to examine the perception of metrical structure, especially under conditions when fewer resources are available (Honing et al., 2014). MMN amplitude indexes the magnitude of a regularity violation (Näätänen et al., 2007) and could therefore function as an index of detectability of the deviants in Experiment 2. In general, an effect of metrical structure on the MMN amplitude in response to deviants would indicate that a metrical structure was induced with lower levels of attentional resources directed at the rhythm. The direction of such an effect could serve as additional evidence to differentiate between temporal attending and temporal prediction. In line with the predictions for Experiment 1, temporal attending was hypothesized to lead to larger MMN amplitudes for deviants in metrically strong than weak positions. Temporal prediction was hypothesized to lead to larger MMN amplitudes for increments in metrically weak than strong positions and for decrements in metrically strong than weak positions.

In addition, the use of EEG allowed us to look at the effects of the metrical structure on auditory evoked potentials at different metrical positions, specifically the P1 and N1. These components are generated in the primary and secondary auditory cortices and have been shown to be sensitive to both attention (Picton and Hillyard, 1974; Woldorff et al., 1993) and prediction (Schafer et al., 1981; Lange, 2009). Whereas enhancement of these components on the beat may be indicative of the presence of temporal attending, attenuation would imply the presence of temporal prediction (Lange, 2013). Thus, in Experiment 2, a possible effect of metrical structure on auditory evoked potentials would provide additional support that a metrical structure was induced with lower levels of attentional resources, with temporal attending leading to enhancement of evoked potentials in response to events in metrically strong positions and temporal prediction leading to attenuation.

Finally, we looked at possible anticipatory effects of temporal attending and prediction, which may be visible before the onset of a stimulus. Indeed, anticipatory processes related to regularity detection have been shown previously using EEG in beta band oscillatory activity (Fujioka et al., 2012). Temporal expectations have also been linked to ERP components, most notably the contingent negative variation (CNV), a negative-going deflection that has been originally associated with the anticipation of a motoric response (Walter et al., 1964). CNV has also been shown to occur in the absence of an overt response (Mento, 2013) and is sensitive to the temporal interval that is anticipated, peaking at the expected time of an event (Praamstra et al., 2006; Mento, 2013). Thus, temporal expectations can be seen in ERPs even before the onset of an event. Therefore, we also looked at possible differences in the ERPs preceding sounds to examine whether we could differentiate between metrical positions on the basis of anticipatory differences.

To summarize, we examined the influence of a perceived metrical structure on the processing of rhythmic events with and without attention directed at the rhythm. We used an isochronous rhythm in which infrequent intensity increments and decrements were introduced to disentangle the contributions of temporal attending and prediction. In the attended condition (Experiment 1), a speeded reaction time task was used to probe the detectability of the deviants. In the unattended condition (Experiment 2), we used the MMN as an index of detectability and additionally looked at the effects of metrical structure on early auditory evoked potentials and anticipatory activity.

## Experiment 1

### Methods

#### Participants

In this experiment we looked at beat perception in an isochronous rhythm. The lack of acoustic cues and the lower attentional resources (cf. Experiment 2) may lead to weaker effects of beat perception (Bouwer et al., 2014). To maximize the chances of inducing a beat under these circumstances we tested only professional musicians. Twenty highly trained musicians (4 males, 16 females) participated in Experiment 1. They were on average 26 years old (range 18–49 years, standard deviation 8 years) and had had an average of 16 years of formal musical training (range 8–23 years, standard deviation 4 years). The instruments they played were clarinet (3), violin (2), viola (1), cello (3), trumpet (1), trombone (2), bassoon (1), flute (1), oboe (1), French horn (2), and piano (1). Two participants were singers. 18 participants were mostly trained and active in classical music, while two participants were trained and active in other genres (pop, world music, jazz). The participants reported an average of 3.3 h of daily practice on their instrument at the time of the experiment (range 1–7 h, standard deviation 1.3 h). All participants provided written informed consent prior to the study. The study was approved by the Ethics Committee of the Faculty of Humanities of the University of Amsterdam.

#### Stimuli

The standard pattern consisted of eight isochronous woodblock sounds with an inter-onset interval of 250 ms (see Figure 1). A binary pattern of subjectively accented and unaccented tones at this rate would put the inter-beat interval at 500 ms, close to the preferred tempo for beat perception (Fraisse, 1982; London, 2002). Patterns were presented in a continuous stream. To prevent participants from shifting the phase of the perceived binary pattern, a click track sound was superposed on the pattern in position 1 (see Figure 1). The time between two click track sounds was 2000 ms (i.e., every eight events). While this may have induced a regular expectation based on acoustic saliency of the click track sound, it is unlikely that people heard a beat at this very slow rate (London, 2002). The woodblock sound was generated in GarageBand (Apple Inc.). The click track sound was 70 ms long, had a MIDI pitch of 74 (587 Hz) and was generated in Audacity (http://audacity.sourceforge.net/). The peak intensity of the click track sound was set to 31 dB lower than the peak intensity of the woodblock sound. Figure 1 (top) shows a schematic representation of the standard stimulus.

In addition to the standard pattern, we generated patterns containing deviants in four different positions (Figure 1, bottom). Two types of deviants were used: intensity increments and intensity decrements. Three different magnitudes of deviants were used: 4, 6, and 9 dB, the smallest being comparable to a subjective accent (Povel and Okkerman, 1981; Brochard et al., 2003). As such, we created a total of 24 different deviant patterns. Deviants were introduced in positions 4, 5, 6, and 7 in the pattern. Previously, using similar stimuli, Bolger et al. (2014) found large effects of metrical expectations in the positions preceding and coinciding with an acoustically salient tone in the first position of an eight-tone pattern. However, as we were specifically not interested in the expectations induced by an exogenous, acoustic cue, we did not use positions 1 and 8, which coincided with and directly preceded the click track sound. In addition, we did not introduce deviants in positions 2 and 3, to avoid confounds due to pattern learning. We have shown that the acoustic context can have a large effect on ERPs in general and MMN in particular, even when difference waves are used (Bouwer et al., 2014; Honing et al., 2014). While difference waves can be used to eliminate the direct effects of acoustic context, the context may have indirect effects on ERPs if a listener has expectations based on the sequential probabilities within a repeating pattern. A deviant in position 2 would have been the only deviant that directly followed the click track sound and as such would have had different sequential properties than the deviants in other positions. While we do not know whether a deviant in position 3 would still be susceptible to this confound, we preferred to err on the side of caution and only introduced deviants in positions 4, 5, 6, and 7 in the pattern.

#### Procedure

Standard patterns and patterns containing a deviant were presented in a continuous stream (see Supplementary Audio). A deviant could occur in 33% of the patterns. As a deviant was only one out of eight tones in a pattern, of the single tones, 4% was a deviant. Of single tones, 83% were standard woodblock sounds, while 13% were click track sounds. Each of the 24 deviant patterns was presented 25 times. Thus, in total 600 deviant and 1200 standard patterns were presented. The experiment was divided into 12 blocks of 5 min, with each block consisting of 50 deviant and 100 standard patterns. Presentation was pseudo-randomized, with the types and magnitudes of the deviants being completely random while there was always at least one standard pattern between two patterns containing a deviant. Participants were instructed to respond with a button press every time they heard something unexpected in the rhythm. Before the experiment started, they were presented with a practice block of 3 min (60 standard and 30 deviant patterns with the same pseudo-randomization as during the experiment) to get familiarized with the task. If needed, they could repeat this practice block until they felt comfortable doing the task. Stimuli were presented through custom-made speakers that were positioned at an angle of 39° and a distance of 132 cm to both sides measured from the back of the chair in which participants were seated. Sound level was set at 60 dB SPL for the standard woodblock sounds, as measured at the back of the chair with a Quest 2800 sound level meter. Presentation® software (Version 14.9, www.neurobs.com) was used to present the stimuli.

#### Analysis

Only responses made between 200 and 1000 ms after presentation of the deviant were included as valid responses. For D1 and D2, this eliminated any responses made after the start of the subsequent pattern. For D3 and D4, this meant responses made after more than 750 and 500 ms respectively were overlapping with the next pattern. For D3, less than 3% of the responses were made after the start of the next pattern. For D4, 29% of responses were made after the start of the next pattern. In the slowest condition at this position (4 dB decrements), 55% of the responses were slower than 500 ms, 85% of the responses were made within 200 ms after the start of the next pattern and 95% were made within 250 ms after the start of the next pattern. As these response times would also have included the motor preparation and response, it is unlikely that they were due to erroneous responses to the next click track sound. Therefore, we did not correct the reaction times beyond the exclusion of reaction times longer than 1000 ms. Average reaction times and miss rates for each condition and each participant were entered into a repeated measures ANOVA with the within subject factors position (D1, D2, D3, and D4), type (increment or decrement) and magnitude (4, 6, or 9 dB difference between the deviant and the standards). We used three orthogonal contrasts to examine possible effects of the position of the deviant. First, to answer our main questions about the contributions of temporal attending and prediction to the processing of metrical rhythm, we compared the responses to deviants on the beat (positions 5 and 7, D2 and D4) with the responses to deviants offbeat (positions 4 and 6, D1 and D3). Second, to examine the possible presence of perceived higher order regularity, we compared the responses to deviants on the third beat (position 5, D2) with the responses to deviants on the fourth beat (position 7, D4). Finally, to check for possible serial position effects, we compared the responses to deviants in the metrically equally weak positions 4 (D1) and 6 (D3). Where applicable, Greenhouse-Geiser corrections were applied to correct for violations of the non-sphericity assumption. The analysis was performed in SPSS Statistics 20.

### Results

**Figure 2:**
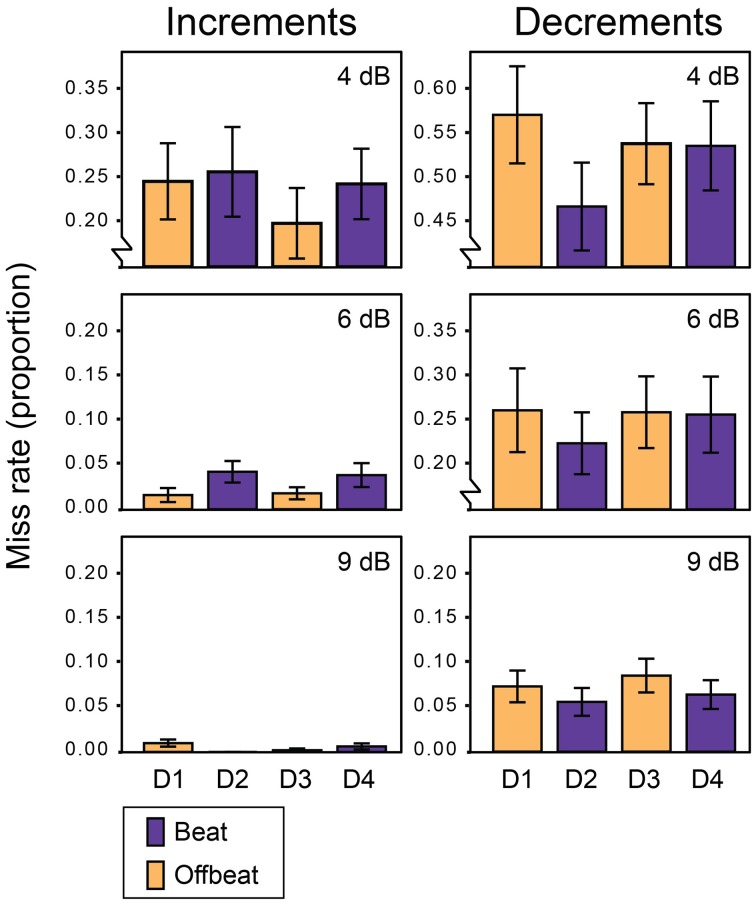
**Miss rates for all deviants in Experiment 1**. Error bars denote one standard error. NB: range of the Y-axis varies between plots for displaying purposes.

**Figure 3:**
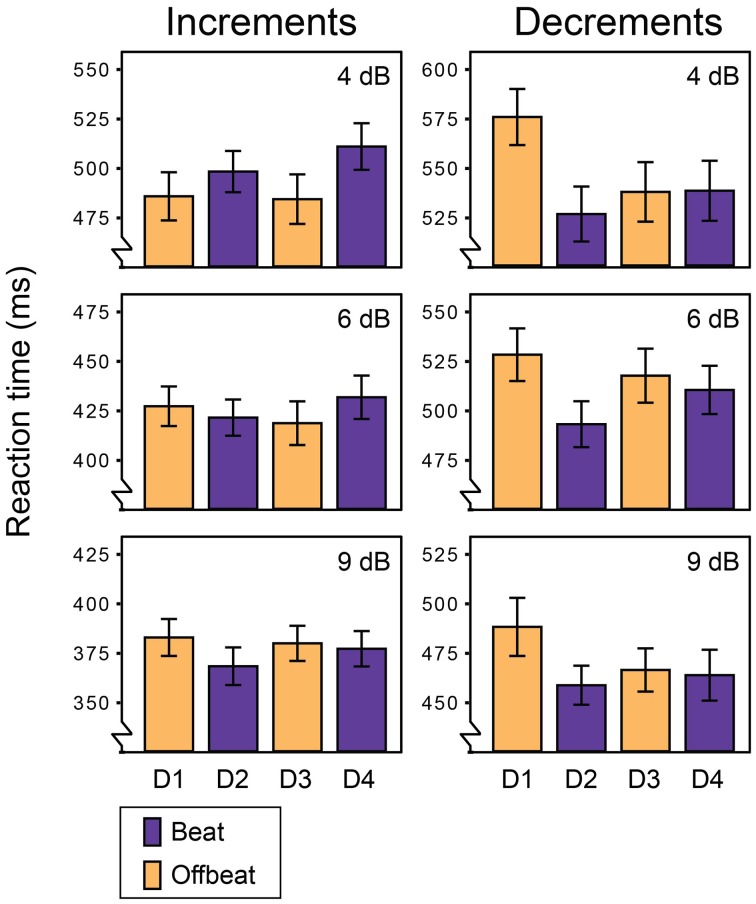
**Reaction times for all deviants in Experiment 1**. Error bars denote one standard error. NB: range of the Y-axis varies between plots for displaying purposes.

Figure 2 shows the average miss rates for beat and offbeat positions and Figure 3 shows the average reaction times. There was a significant interaction between deviant type and metrical position for both miss rates [*F*_(3, 57)_ = 6.1, *p* = 0.001, η^2^ = 0.24] and reaction times [*F*_(3, 57)_= 10.7, *p* < 0.001, η^2^ = 0.36]. Therefore, we ran additional ANOVAs for increments and decrements separately. For decrements, miss rates were affected by both position [*F*_(3, 57)_ = 4.9, *p* = 0.004, η^2^ = 0.20] and magnitude [*F*_(2, 38)_= 134.1, *p* < 0.001, η^2^ = 0.88] of the deviant. Decrements on the beat (D2 and D4) were detected more often than decrements offbeat [D1 and D3; *F*_(1, 19)_ = 15.4, *p* = 0.001, η^2^ = 0.45]. In addition, decrements on the strong beat in position 5 (D2) were detected more often than decrements on the weaker beat in position 7 [D4; *F*_(1, 19)_ = 4.6, *p* = 0.045, η^2^ = 0.20]. Reaction times showed a similar pattern of results, with significant effects of position [*F*_(3, 57)_ = 10.6, *p* < 0.001, η^2^ = 0.36] and magnitude [*F*_(2, 38)_ = 57.1, *p* < 0.001, η^2^ = 0.75]. Decrements on the beat were detected faster than decrements offbeat [*F*_(1, 19)_ = 17.1, *p* = 0.001, η^2^ = 0.47]. Finally, decrements in position 6 (D3) were detected faster than decrements in position 4 [D1; *F*_(1, 19)_ = 13.7, *p* = 0.002, η^2^ = 0.42]. As this may indicate a serial position effect either hindering detection of D1 or facilitating detection of D3, we performed additional post-hoc contrasts comparing the reaction time for D3 to the reaction times for D2 and D4 separately. While the difference between the reaction times to D2 and D3 was significant [*F*_(1, 19)_ = 8.4, *p* = 0.009, η^2^ = 0.31], the comparison between D3 and D4 was not (*F* < 0.3).

For increments, miss rates were also affected by both position [*F*_(3, 57)_ = 3.6, *p* = 0.020, η^2^ = 0.16] and magnitude of the deviant [*F*_(2, 38)_ = 33.2, *p* < 0.001, η^2^ = 0.64]. Contrary to decrements, increments were detected more often offbeat (D1 and D3) than on the beat [D2 and D4; *F*_(1, 19)_= 9.9, *p* = 0.005, η^2^ = 0.34]. In addition, increments were detected more often in position 6 (D3) than in position 4 [D1; *F*_(1, 19)_ = 4.9, *p* = 0.039, η^2^ = 0.21], which may indicate a similar serial position effect as found for reaction times to decrements. To check whether this may have driven the difference in detection rate between increments on the beat and offbeat, we performed post-hoc tests contrasting the miss rates for D3 with those for D2 and D4. Both comparisons were significant, indicating better detection of increments in position 6 (D3) than positions 5 and 7 [D2; *F*_(1, 19)_ = 9.5, *p* = 0.006, η^2^ = 0.33 and D4; *F*_(1, 19)_ = 6.9, *p* = 0.017, η^2^ = 0.27].

For reaction times to increments, there was a significant interaction between the position and magnitude of the deviant [*F*_(6, 114)_ = 2.8, *p* = 0.046, η^2^ = 0.13]. To look at the nature of the interaction effect, we ran ANOVAs for each magnitude separately. The reaction times for small (4 dB) and large (9 dB) increments were significantly affected by the position of the deviant [*F*_(3, 57)_ = 3.0, *p* = 0.037, η^2^ = 0.14 and *F*_(3, 57)_ = 4.1, *p* = 0.011, η^2^ = 0.18 respectively]. However, metrical position had opposite effects on the detection of small and large increments. Small increments were detected faster offbeat than on the beat [*F*_(1, 19)_ = 8.4, *p* = 0.009, η^2^ = 0.31], while large increments were detected faster on the beat than offbeat [*F*_(1, 19)_ = 13.4, *p* = 0.002, η^2^ = 0.41]. Position did not affect reaction times for 6 dB increments. Finally, 9 dB increments on the strong beat in position 5 (D2) were detected marginally faster than increments on the weaker beat in position 7 [D4; *F*_(1, 19)_ = 3.9, *p* = 0.062, η^2^ = 0.17].

### Discussion

The results from Experiment 1 suggest that temporal prediction and temporal attending, as well as an interaction between them mediate the effect of metrical position on the perception of rhythmic events. The influence of temporal prediction is apparent from faster and better detection of small increments offbeat than on the beat (see Table 1), likely due to the prediction error being larger for increments offbeat than on the beat. The influence of temporal attending is apparent from faster detection of large increments on the beat than offbeat, likely due to heightened sensitivity for events on the beat. The effects of temporal prediction thus seem to be counteracted by temporal attending for large but not small increments. This cannot be explained by assuming additivity of both mechanisms, but instead shows an interaction. Previously, it has been suggested that attention may act to boost the precision of the prediction error (Feldman and Friston, 2010; Kok et al., 2012). For small increments, the prediction error on the beat was likely very small or even absent, as an increment of this size is comparable in magnitude to a subjective accent (Povel and Okkerman, 1981; Brochard et al., 2003). The weighted prediction error for small increments, taking into account a boost from heightened attentional resources on the beat but not offbeat, was likely still smaller on the beat than offbeat. As the prediction error for large increments would have been substantially bigger, it would have benefitted more from a boost from heightened attentional resources on the beat and this would have outweighed the larger prediction error for increments in offbeat positions. The results for increments as such are consistent not only with the presence of both temporal prediction and temporal attending but also with an interaction between these mechanisms in which attention boosts the precision of predictions. Decrements, as expected, were detected better and faster on the beat than offbeat, which is in line with both temporal prediction and temporal attending.

In addition to differences between the detection of deviants on the beat and offbeat, we also found effects of meter and serial position. Decrements were detected more often and large increments marginally faster on the strong third beat (position 5) than on the weaker fourth beat (position 7), consistent with heightened sensitivity for events in metrically strong positions and thus with temporal attending driving this effect of meter. A serial position effect was apparent from faster detection of decrements and better detection of increments in position 6 than in position 4, while these positions were metrically equally weak. Possibly, the temporal proximity of deviants in position 4 to the click track sound made them harder to detect. When not taking into account position 4, which may have been biased, our post-hoc contrasts show that decrements on the third beat (position 5) were detected faster than decrements offbeat (position 6) and increments were detected better offbeat (position 6) than on the beat (positions 5 and 7). As such, the observed effects of temporal attending and prediction cannot be explained solely by the presence of a serial position effect.

While the results of Experiment 1 do not allow us to estimate the relative contribution of the two mechanisms involved, we showed that temporal attending, temporal prediction and an interaction between them influence the processing of rhythmic events within a metrical structure. In Experiment 2, using EEG, we examined whether the same mechanisms would be present with lower general levels of attention resources devoted to the rhythm.

## Experiment 2

### Methods

#### Participants

Twenty-four highly trained musicians (8 males, 16 females) participated in Experiment 2, 12 of whom had also participated in Experiment 1. Their average age was 28 years old (range 19–58 years, standard deviation 8 years) and they had received an average of 19 years of formal musical training (range 7–46 years, standard deviation 8 years). The instruments this group of participants played were clarinet (3), violin (5), cello (3), trumpet (1), bassoon (1), flute (2), guitar (2), French horn (3), and piano (3). One participant was a singer. Twenty-two participants were mostly trained and active in classical music, while two participants were trained and active in other genres (pop, world music, jazz). They reported an average of 3.1 h of daily practice on their instrument at the time of the experiment (range 1–5 h, standard deviation 1.1 h).

#### Stimuli

The stimuli were largely the same as those used in Experiment 1 (see Figure 1). However, due to time constraints imposed by the use of EEG we only used deviants of 9 dB, as we expected large deviants to elicit a reliable MMN. Deviants were, similar to Experiment 1, either increments or decrements and were introduced at positions 4, 5, 6, and 7 in the rhythm. In total, we thus used eight deviant patterns. The peak amplitude of the click track sound in Experiment 2 was set to 10 dB lower than the peak intensity of the woodblock sound to ensure participants heard the metrical structure with the same phase alignment under unattended conditions.

#### Procedure

Increments and decrements were tested in separate sessions using 150 deviants on each of the eight possible positions, resulting in a total of 600 deviant patterns for each type. Deviant patterns represented 33% of the total patterns, with deviant tones making up 4% of total sounds. Thus, a total of 1800 patterns was presented in each session. Patterns were presented in five blocks of 12 min (360 patterns), presented in a continuous stream. As in Experiment 1, patterns were presented in pseudo-randomized order, with at least one standard pattern between two patterns containing a deviant. To minimize possible effects of short-term learning of the rhythmic pattern during the attended behavioral task, those participants that participated in both Experiment 1 and Experiment 2 participated in the EEG task either preceding the behavioral task or on a different day. During the presentation of the rhythms participants watched a self-selected silenced movie with subtitles. They were instructed to concentrate on the movie and to ignore the rhythm. All participants indicated that they could comply with this task. Each condition took around 1 h to complete. Participants could take breaks as needed. The sound equipment was identical to Experiment 1.

#### EEG recording

EEG was recorded with a 64-channel Biosemi Active-Two reference free acquisition system (Biosemi, Amsterdam, The Netherlands), using the standard 10/20 configuration and additional electrodes at both mastoids, around the eyes and on the nose. The EEG signal was recorded at 8 kHz.

#### EEG analysis

EEG preprocessing was performed in Matlab (Mathworks, Inc.) using the EEGLAB toolbox (Delorme and Makeig, 2004). The statistical analysis was performed in SPSS Statistics 20. For all analyses described below, where applicable Greenhouse-Geiser corrections for non-sphericity were used. For the analysis of ERP responses to both deviants and standards, EEG data was offline re-referenced to linked mastoids and down-sampled to 512 Hz. In eleven participants, one or more bad channels was removed and subsequently interpolated from the surrounding channels. None of these channels is reported here. Independent component analysis was used to remove eye-blinks.

##### Analysis of ERP responses to deviants

For the analysis of the MMN, data were filtered between 0.5 and 20 Hz, using a linear finite impulse response filter and 650 ms epochs were extracted from the continuous data starting 150 ms before the onset of each deviant. Epochs at the same positions were extracted from the standard patterns. Epochs with an amplitude difference of more than 100 microvolts within a 500 ms sliding window were rejected from the analysis, epochs were averaged for each condition separately and baseline corrected using the average activity of the 150 ms pre-stimulus period. Deviant-standard difference waves were calculated by subtracting the ERP obtained in response to the standards from the ERP in response to the deviants aligned in time relative to the start of the pattern. We defined the MMN as the negative peak between 100 and 200 ms after the onset of the deviant. Visual inspection of the group averaged difference waves for the different conditions showed a large difference in morphology between the responses to increments and decrements. To quantify this difference, we performed an analysis of the peak latencies of the MMN at electrode Fz (see Table 2). Peak latencies for all participants for all deviants were entered into an ANOVA with factors type (increments and decrements) and position (D1, D2, D3, and D4). The type of deviant significantly affected the peak latency, with later peaks for decrements than increments [*F*_(1, 23)_ = 11.4, *p* = 0.003, η^2^ = 0.33]. No effects of position on peak latencies was observed, nor an interaction between type and position.

**Table 2:** **Mean average peak latencies and average amplitudes of the MMN to deviants**.

	**Average peak latency (ms)**		**Average amplitude (μV)**	
	**Increments**	**Decrements**	**Increments**	**Decrements**
D1	157 (32)	165 (27)	−0.52 (1.47)	−0.93 (1.41)
D2	140 (27)	165 (23)	−0.26 (1.19)	−1.44 (1.10)
D3	148 (30)	161 (27)	−0.97 (1.50)	−1.48 (1.13)
D4	149 (31)	164 (30)	0.11 (1.70)	−1.14 (1.18)

**Figure 4:**
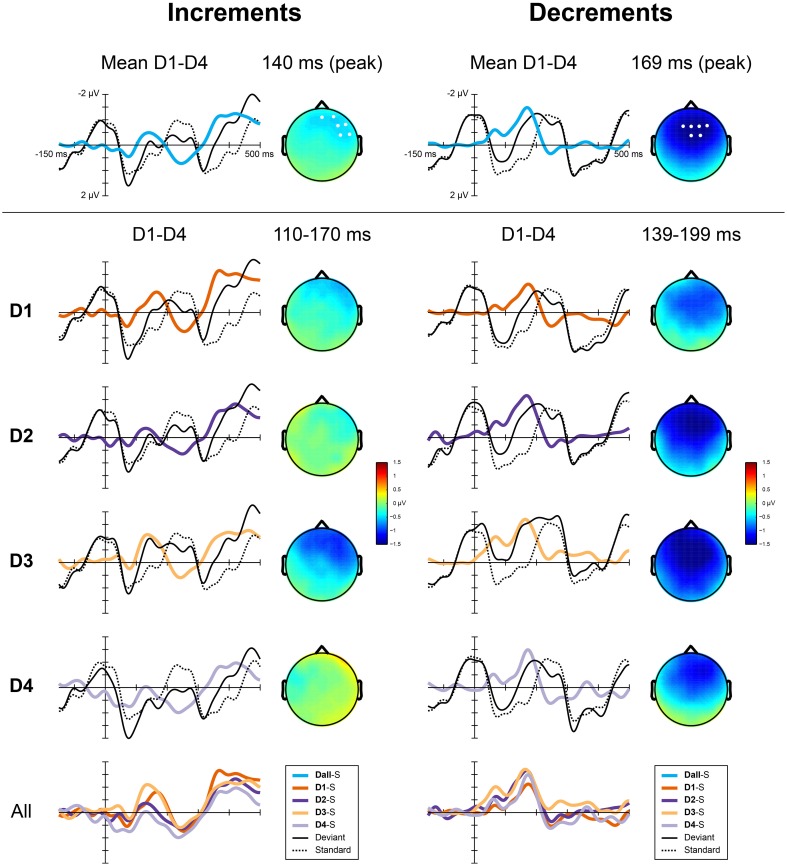
**ERP responses to the deviants in Experiment 2 for increments (left) and decrements (right)**. Top panels show the difference waves for both types collapsed over positions, the scalp distributions at the peak latency of the MMN and the regions of interest used for the analysis. Middle panels show, for each position separately, group averaged ERPs elicited by the deviants, the standards (S), the derived difference waves and the scalp distribution of the MMN averaged over the analysis window. The bottom panel shows all difference waves combined.

*Peak latencies are the negative peak between 100 and 200 ms on Fz. Amplitudes are as used for the analysis, measured on ROIs as specified in Figure 4 from a 60 ms window around the peak for the averaged increments and decrements separately. Standard deviations in brackets*.

A difference between the responses to increments and decrements has previously been observed (Rinne et al., 2006) and may be due to overlap with other ERP components that are affected by the intensity of the deviants. As the responses to the different deviant types were qualitatively different, we performed the statistical analysis separately for increments and decrements. We calculated the average difference waves for increments and decrements collapsed over the four metrical positions. These difference waves are shown in Figure 4 (top). The MMN for increments peaked at a latency of 140 ms, while the MMN for decrements peaked at 169 ms. At the peak latency, the MMN for increments showed a right-frontal scalp distribution, while the MMN for decrements was slightly more centrally located. For both types, we defined a region of interest for the analysis of the MMN encompassing the 6 electrodes with highest amplitudes at the peak latency. These regions of interest are indicated in Figure 4 (top).

For the analysis, the MMN amplitude was defined as the average amplitude in a 60 ms window around the peak of the MMN for each type collapsed over positions. As such, we defined the window for analysis independent from the metrical positions, while acknowledging the differences due to the different types of deviants. MMN amplitudes were entered into a repeated measures ANOVA with the within subject factor position (D1, D2, D3, and D4). The same contrasts as in Experiment 1 were used to explore the effect of the position of the deviant on the MMN amplitude. To examine the effect of metrical structure, the responses to deviants in offbeat positions (D1 and D3) were compared to the responses to deviants on the beat (D2 and D4). To examine the possible presence of higher order regularity in the form of meter, we compared the response to deviants on the third beat (D2) to the response to deviants on the, theoretically less salient, fourth beat (D4). Finally, to look at possible serial position effects, we compared the responses to deviants in positions 4 and 6 (D1 and D3), which were both metrically weak.

##### Analysis of ERP responses to standards

Regarding the analysis of AEPs in response to the standards, we were mainly interested in the P1 and N1 components. To optimize the analysis of the standards to these shorter latency components, we filtered the data using linear finite impulse response filtering between 5 and 75 Hz (see Schwartze et al., 2013, for a discussion of these filter settings). Epochs starting at 50 ms before the onset of each sound in the standard patterns and ending at 250 ms after the onset of each sound were extracted from the continuous data. Epochs with an amplitude difference larger than 150 microvolts were rejected and epochs were averaged for each position separately to obtain ERPs. ERPs were averaged over blocks of deviant types, as the standards were exactly the same in both conditions. No baseline correction was applied. With a stricter high-pass filter, the effects of slow amplitude changes are much less pronounced, making baseline correction unnecessary. Also, while for the MMN analysis we were interested in the reaction to the deviants, which starts the moment the deviant sound is heard, for the analysis of the standards, we were also interested in possible differences in anticipatory activity. If these effects would indeed be present, a baseline correction would falsely eliminate any differences between conditions, while possibly falsely creating differences between conditions in the P1 or N1 responses due to differences in the baseline.

The amplitude of the P1 and N1 was defined as the average amplitude in a 40 ms window around the average latency of the peaks of these components for all four positions. The peak latency of the P1 response was 63 ms and the peak latency of the N1 response was 133 ms. For anticipatory activity, the 40 ms window was centered around 0, where anticipatory activity was expected to be maximal. Statistical analysis was thus conducted for three time windows: 43–83 ms for P1, 113–153 ms for N1 and −20–20 ms to look at differences in anticipatory activity. For the analysis of the standards, we used a region of interest containing fronto-central midline electrodes (Cz, FCz, and Fz). Like for the deviants, we only included the ERPs in response to sounds in positions 4–7 in the analysis, to avoid confounds due to the click track sound. We tested the same orthogonal contrasts as described for the analysis of the MMN.

### Results

#### ERP responses to deviants

Figure 4 (bottom) shows the difference waves for all deviants. Table 2 shows the average amplitudes and peak latencies for all conditions. For increments, we found a marginal effect of metrical position, with a larger amplitude MMN offbeat than on the beat [*F*_(1, 23)_ = 3.0, *p* = 0.097, η^2^ = 0.12]. In addition, the MMN to increments on the strong third beat (D2) was marginally larger than the MMN to increments on the weaker fourth beat [D4; *F*_(1, 23)_ = 2.9, *p* = 0.10, η^2^ = 0.11]. For decrements, the MMN to deviants on position 4 (D1) was smaller than the MMN to deviants on position 6 [D3; *F*_(1, 23)_ = 5.6, *p* = 0.026, η^2^ = 0.20], possibly indicating a serial position effect.

#### ERP responses to standards

**Figure 5:**
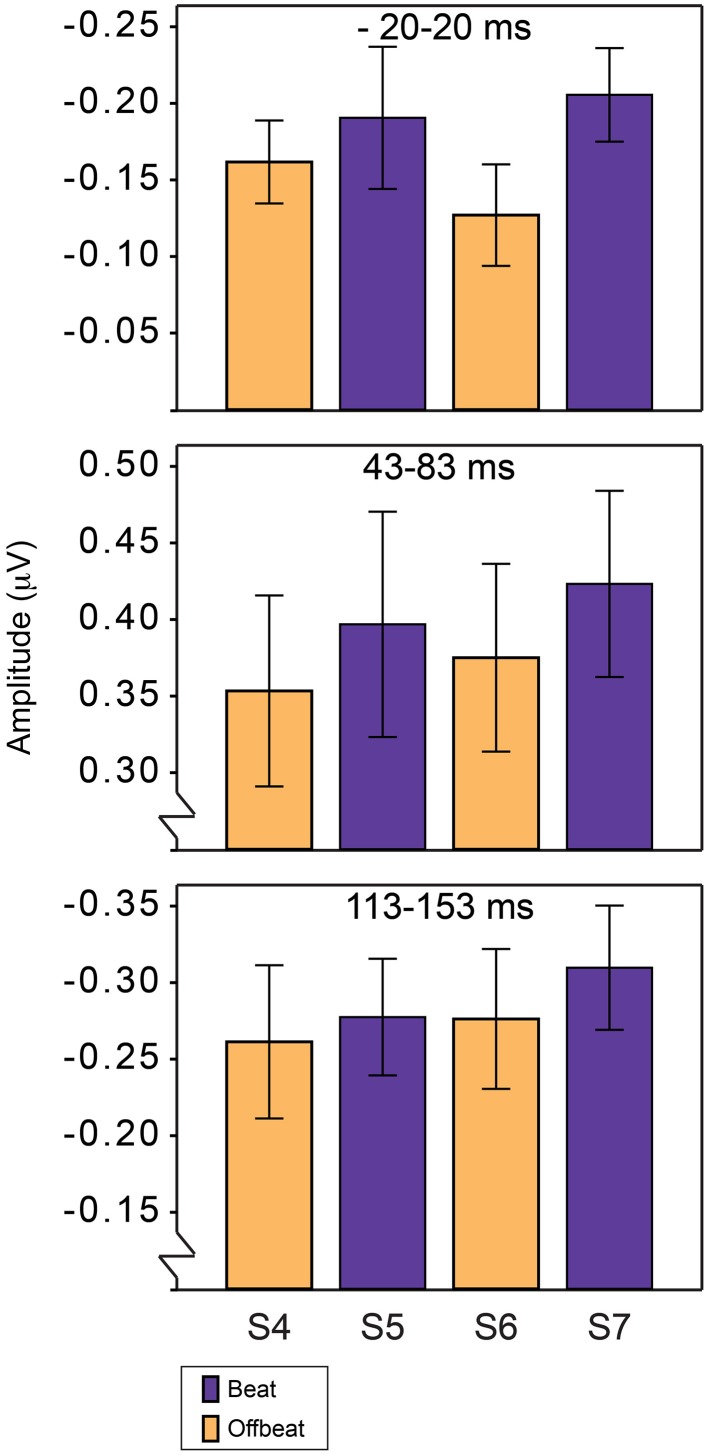
**Average magnitudes of ERP components in response to standards on the beat and offbeat in Experiment 2**. Anticipatory negativity **(top)**, P1 **(middle)**, and N1 **(bottom)**. Responses are shown for positions 4–7 in the standards, corresponding to the positions in which deviants D1–D4 could occur.

**Figure 6:**
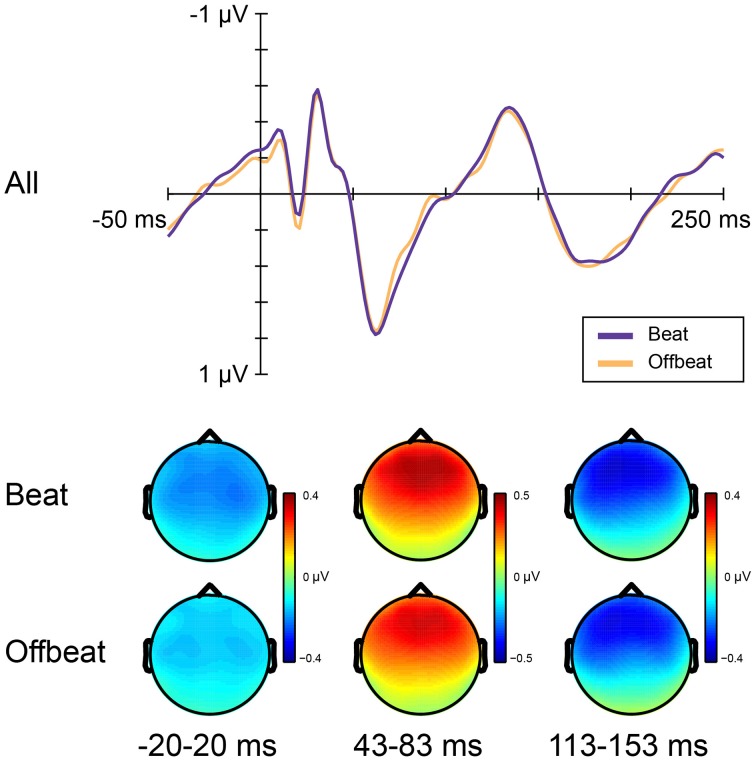
**ERP responses elicited by standards on the beat and offbeat**. **Top** panel shows ERPs collapsed over metrical position (on the beat: positions 5 and 7; offbeat: positions 4 and 6). **Bottom** panel shows scalp distributions for analysis windows.

Figure 5 shows the average amplitudes for all positions in the standard pattern of all time windows of interest. Table 3 lists the average amplitudes and peak latencies. ERPs for positions 4–7, collapsed over metrical levels, are shown in Figure 6. Around the baseline, the anticipatory activity was more negative for sounds on the beat than offbeat [*F*_(1, 23)_ = 5.2, *p* = 0.033, η^2^ = 0.18]. The P1 amplitude was larger on the beat than offbeat [*F*_(1, 23)_ = 4.30, *p* = 0.049, η^2^ = 0.16]. None of the other contrasts was significant.

**Table 3:** **Mean average peak latencies and average amplitudes of the ERP responses to standards**.

	**Anticipatory**	**P1**		**N1**	
	**Average amplitude (μV)**	**Average peak latency (ms)**	**Average amplitude (μV)**	**Average peak latency (ms)**	**Average amplitude (μV)**
S4	−0.16 (0.13)	72 (16)	0.35 (0.31)	129 (18)	−0.26 (0.25)
S5	−0.19 (0.23)	71 (14)	0.40 (0.36)	132 (15)	−0.28 (0.19)
S6	−0.13 (0.16)	65 (11)	0.38 (0.30)	127 (16)	−0.28 (0.22)
S7	−0.21 (0.15)	66 (12)	0.42 (0.30)	130 (13)	−0.31 (0.20)

*For the anticipatory negativity, amplitudes are as measured from a 40 ms window around the onset of the sound. We do not report peak latencies for this component as we cannot estimate the peak from our data. Peak latencies for P1 are defined as the positive peak between 40 and 100 ms on midline electrodes. Peak latencies for N1 are defined as the negative peak between 100 and 180 ms on midline electrodes. Amplitudes are as used for the analysis, measured on midline electrodes as specified in Figure 6 from a 40 ms window around the peak for each component averaged over conditions. Standard deviations in brackets*.

### Discussion

The results of Experiment 2 regarding the responses to deviants suggest that even with lower levels of attentional resources available for the perception of a rhythm, temporal prediction and temporal attending affect processing of regular rhythmic events. The MMN amplitude for intensity increments was marginally larger offbeat than on the beat. This is in line with a larger prediction error for increments offbeat than on the beat and thus suggests the presence of temporal prediction. In addition, the MMN amplitude for increments on the strong third beat was marginally larger than for increments on the weaker fourth beat. This is in line with heightened sensitivity for events in metrically salient positions and thus suggests the presence of temporal attending. However, the results for the deviants are tentative at best, with no effect of metrical position on the MMN responses to intensity decrements, and only marginally significant effects of metrical position on the MMN responses to increments. The latter may be due to the effects of temporal attending and temporal prediction canceling each other out. However, for decrements, the simultaneous presence of both mechanisms should have strengthened the results. Also, like in Experiment 1, serial position effects could be observed for decrements, with smaller responses to decrements in position 4 than in position 6. As such, we have to be cautious in interpreting the findings regarding the influence of metrical position on MMN amplitude.

The results of Experiment 2 regarding the responses to standards provide additional support for the presence of temporal attending. The P1 response was larger for events on the beat than offbeat, consistent with the results of Tierney and Kraus (2013). This enhancement of the response to sounds on the beat may be due to attention peaking at metrically strong moments in time and leading to enhancement of early sensory processing (Lange, 2013). We did not find any effect of metrical position on the amplitude of the N1. A similar enhancement due to attention of the P1 but not the N1 has been reported previously (Karns and Knight, 2008; Tierney and Kraus, 2013). However, the opposite effects, attenuation of the P1 and enhancement of the N1, have also been shown simultaneously in a study manipulating the temporal predictability of auditory events (Rimmele et al., 2011). These different results are likely due to differences in stimuli and tasks that influenced the relative contributions of temporal attending and prediction.

Finally, in anticipation of standard events on the beat, ERPs were more negative than in anticipation of standard events offbeat. The fact that this difference was present at the onset of the events and that the activity for more expected events (on the beat) was negative relative to the activity for less expected events (offbeat) makes it reminiscent of the contingent negative variation (CNV; Walter et al., 1964), a negative-going ERP component peaking at the expected time of an event. Whether the processes underlying the CNV are relevant to the perception of a metrical structure is unclear, but our results show that it may be fruitful to acknowledge possible differences in brain activity preceding the onset of events when examining the perception of metrical rhythm using ERPs.

One final remark must be made about the ERP results. While all participants reported being able to focus on the movie during the experiment, we cannot completely rule out that the results we found are due to lapses in attention. We feel confident that participants were listening to the rhythms with lower levels of attentional resources while watching the movie than while performing a task on the rhythm itself. However, to draw stronger conclusions about the influence of attentional resources on the perception of metrical structure, results with and without attention directed at the rhythm should be acquired using the same method. Furthermore, to be able to prevent and control for attentional lapses a continuous task should be used to direct attention away from the rhythm. Within the context of EEG research, this provides practical challenges that future experiments will have to tackle.

## General discussion

We have shown that the induced metrical structure influences the processing of rhythmic events through the influence of both temporal attending and temporal prediction. Moreover, our data suggest that both temporal attending and prediction are involved in processing of metrical rhythm when attention is directed away from the rhythm. Temporal attending was apparent from heightened sensitivity for events in strong metrical positions. Unexpected intensity decrements and large increments were detected better and faster on the beat than offbeat and decrements were detected better on the strong third beat than on the weaker fourth beat (Experiment 1). In addition, the auditory P1 for standard events on the beat was enhanced and the MMN amplitude for increments was marginally larger on the strong third beat than on the weaker fourth beat (Experiment 2). Temporal prediction was apparent from better detection of events that elicited a large prediction error. Small increments were detected faster and better offbeat than on the beat (Experiment 1) and the MMN amplitude for increments on the beat was marginally larger than for increments offbeat (Experiment 2). Finally, an interaction between temporal attending and prediction was evident from the interaction between the magnitude of the deviant and the effect of metrical position in Experiment 1. This interaction is in line with temporal attention boosting the precision and the weighting of the prediction error (Kok et al., 2012).

The complex interplay of temporal attending and temporal prediction may explain previous conflicting findings regarding the processing of metrical rhythm. While some studies found enhancement of early sensory processing in metrically strong positions (Tierney and Kraus, 2013), others found attenuation (Schwartze et al., 2013). Interestingly, while the former study used real music, and as such had stimuli with presumably multiple levels of regularity present, the latter used isochronous sequences. Arguably, while this tests regularity detection, it is not necessarily examining metrical structure, which by nature has a hierarchical component (Fitch, 2013; Vuust and Witek, 2014). In the current study, consistent with temporal attending, we found enhancement of the auditory P1 in metrically strong positions. We compared responses on the beat with responses offbeat, which constitute different levels in a metrical hierarchy. At a higher level, the differences in responses to deviants on the strong third and weak fourth beat were also consistent with heightened sensitivity for events in metrically strong positions and thus with temporal attending. Possibly, temporal attending plays a relatively larger role than temporal prediction in shaping our perception when different hierarchical levels are used. This would fit nicely with a neural resonance account of metrical perception, which presumes that multiple emergent oscillators cause dynamic fluctuations in attentional resources and the perception of regularity at multiple hierarchical levels (Large, 2008).

Several other factors may influence the relative contributions of temporal attending and prediction on the processing of metrical rhythm. First, it has been suggested that temporal attending is an endogenously driven process, while temporal prediction is driven by bottom-up cues (Sanabria and Correa, 2013). While we found evidence of both processes using stimuli that required mainly endogenous generation of the metrical structure, it is possible that the relative contribution of temporal prediction would be bigger when using stimuli with more exogenous cues indicating the metrical structure. Second, the balance between temporal attending and prediction may be affected by the amount of resources available for processing a rhythm. With the current design, we cannot compare the results of the attended behavioral experiment and the unattended EEG experiment directly. Third, different ERP components may be affected differently by temporal attending and prediction. MMN has been specifically linked to predictive coding (Winkler and Czigler, 2012), and may therefore be more sensitive to the effects of temporal prediction than temporal attending. Also, in the current study, the effect of metrical structure on the amplitude of the auditory P1 but not the N1 may indicate a difference in the sensitivity of these components to temporal attending and prediction. This would also explain the inconsistent findings for these components in previous studies (Rimmele et al., 2011; Schwartze et al., 2013; Tierney and Kraus, 2013).

The effects of temporal attending and prediction we found in Experiment 2, with lower levels of attentional resources directed at the rhythm, were very small, despite the high level of musical expertise of our participants. Previously, we have shown that musically untrained individuals can induce a metrical structure from a rhythm with clear acoustic accents even with lower levels of attentional resources (Bouwer et al., 2014). Whether musical training is necessary to induce a metrical structure from stimuli without acoustic accents under these circumstances remains to be tested. However, as we have shown here that multiple processes contribute to the processing of metrical rhythm, it may be fruitful to look at the influence of musical training on temporal attending and prediction separately. Possibly, temporal attending is a process arising from the properties of the brain itself (Large, 2008) and as such independent of musical training, while temporal prediction relies more on long term learning of musical structure (Vuust and Witek, 2014) and thus may be more susceptible to musical training. As such, temporal predictions may in fact be derived from the perceptual effects of temporal attending. The relationship between temporal attending and prediction and whether musical training, attentional resources and the presence of hierarchy and exogenous cues in a rhythm indeed affect their relative contributions to the processing of metrical rhythms is an interesting topic for future studies.

## Conclusion

We provided evidence in support of concurrent effects of both temporal attending and temporal prediction on the processing of metrical rhythm. This was shown both in an attended behavioral task and in an EEG experiment with attention directed away from the rhythm. These mechanisms can provide useful notions in decomposing the top-down influence of a metrical structure on the processing of rhythm. This opens up interesting possibilities for future work, which should take into account that the perception of metrical rhythm is not simply one process. In addition, the relationship between these processes may inform us about mechanisms underlying the human ability to perceive a metrical structure in musical rhythm, which while being a fundamental aspect of music cognition (Honing et al., 2015), is still ill understood.

### Conflict of interest statement

The authors declare that the research was conducted in the absence of any commercial or financial relationships that could be construed as a potential conflict of interest.

## Footnotes

^1^The use of the term attention in the context of beat perception can lead to confusion (Henry and Herrmann, 2014) as it denotes both the general attentional resources available, usually manipulated by task-relevance of a rhythm and independent from the metrical structure, and the local fluctuations in attentional resources, which, according to DAT, depend on the metrical structure. Here, for the latter we will use the term temporal attending to differentiate it from the general use of the term attention.
